# The association between state bans on soda only and adolescent substitution with other sugar-sweetened beverages: a cross-sectional study

**DOI:** 10.1186/1479-5868-12-S1-S7

**Published:** 2015-07-27

**Authors:** Daniel R Taber, Jamie F Chriqui, Renee Vuillaume, Steven H Kelder, Frank J Chaloupka

**Affiliations:** 1 Institute for Health Research and Policy, University of Illinois at Chicago,1747 West Roosevelt Road, Chicago, IL 60608 Chicago, IL, USA; 2 Michael & Susan Dell Center for Healthy Living, The University of Texas School of Public Health Austin Regional Campus, 1616 Guadalupe St. Austin, TX 78701, USA; 3 Department of Health Policy and Administration, University of Illinois at Chicago, Chicago, IL, USA; 4 Harvard University John F. Kennedy School of Government, 79 John F. Kennedy Street, Cambridge, MA 02138, USA; 5 Department of Economics, University of Illinois at Chicago, Chicago, IL, 601 South Morgan UH725 M/C144, Chicago, IL 60607, USA

**Keywords:** sugar-sweetened beverages, school nutrition, nutrition policy, substitution, adolescents

## Abstract

**Background:**

Across the United States, many states have actively banned the sale of soda in high schools, and evidence suggests that students’ in-school access to soda has declined as a result. However, schools may be substituting soda with other sugar-sweetened beverages (SSBs), and national trends indicate that adolescents are consuming more sports drinks and energy drinks. This study examined whether students consumed more non-soda SSBs in states that banned the sale of soda in school.

**Methods:**

Student data on consumption of various SSBs and in-school access to vending machines that sold SSBs were obtained from the National Youth Physical Activity and Nutrition Study (NYPANS), conducted in 2010. Student data were linked to state laws regarding the sale of soda in school in 2010. Students were cross-classified based on their access to vending machines and whether their state banned soda in school, creating 4 comparison groups. Zero-inflated negative binomial models were used to compare these 4 groups with respect to students’ self-reported consumption of diet soda, sports drinks, energy drinks, coffee/tea, or other SSBs. Students who had access to vending machines in a state that did not ban soda were the reference group. Models were adjusted for race/ethnicity, sex, grade, home food access, state median income, and U.S. Census region.

**Results:**

Students consumed more servings of sports drinks, energy drinks, coffee/tea, and other SSBs if they resided in a state that banned soda in school but attended a school with vending machines that sold other SSBs. Similar results were observed where schools did not have vending machines but the state allowed soda to be sold in school. Intake was generally not elevated where both states and schools limited SSB availability – i.e., states banned soda and schools did not have SSB vending machines.

**Conclusion:**

State laws that ban soda but allow other SSBs may lead students to substitute other non-soda SSBs. Additional longitudinal research is needed to confirm this. Elevated SSB intake was not observed when both states and schools took steps to remove SSBs from school.

## Background

For decades, diet patterns around the world have shifted toward greater consumption of sugar-sweetened beverages (SSBs) [1]. Some of the most striking trends have occurred in the United States (U.S.), where the proportion of daily energy intake that came from SSBs increased from 4.8% to 10.3% from 1977-78 to 1999-2001 [2]. The proportion was even higher among 12- to 19-year-olds, who consumed 13.5% of their energy from SSBs in 1999-2000 [3]. By 2005-06, soda alone accounted for more energy than any other food/beverage group among 14- to 18-year-olds (approximately 946 kJ/day) [4]. Overall SSB consumption has declined in the past decade [3,5], but 12- to 19 year-olds still consumed 10.5% of their energy from SSBs in 2009-10 [3].

These trends have become a prominent public health concern due to the large volume of evidence that SSB consumption is associated with increased weight gain and cardio-metabolic risk factors [6-8]. In response to these concerns, state and local policymakers in the U.S. have aggressively targeted adolescent soda consumption, often by banning the sale of soda in schools. School district policies that ban soda increased rapidly starting in 2006-2007 [9], when districts that participated in federal school meal programs were required to design a wellness policy to target nutrition and physical activity [10]. Several studies have since reported that schools were less likely to sell soda if their state or district had a policy banning soda sales in schools [11-14]. Overall, the proportion of high school students in the U.S. who could purchase soda in school was cut in half in a 5-year span (from 53.6% in 2006-2007 to 25.3% in 2010-2011) [15].

Trends in adolescent soda consumption reversed during this time, as adjusted mean intake decreased from approximately 1,255 to 1,046 kJ/day from 1999-2000 to 2007-2008 [5]. Evidence also suggests, however, that adolescents may be replacing soda with other SSBs. The decrease in soda consumption was balanced by a nearly equal increase in adjusted mean consumption of energy drinks and sports drinks (approximately 531 to 699 kJ/day) [5]. Most countries have not measured beverage-specific trends as extensively, but a study in South Korea also reported that adolescent soda intake was stable from 2001 to 2009, whereas intake from miscellaneous SSBs, including sports/energy drinks, increased [16].

Adolescents often perceive these beverages as healthy alternatives to soda [17] and beverage companies advertise them as such [18]. However, sports drink consumption has been actively discouraged by the American Academy of Pediatrics (AAP) because of sports drinks’ high calorie and sugar content [19], and energy drink consumption has been strongly discouraged by the AAP [19], American Medical Association [20], and International Society of Sports Nutrition [21] because of energy drinks’ high caffeine content and understudied additives such as taurine and guarana [22]. Few countries carefully track adverse health events that are tied to energy drink consumption, but several adverse events have been reported in Germany and New Zealand [22]. Some countries, including Turkey and Uruguay, have banned energy drinks entirely [22].

Policymakers in the U.S. have not targeted non-soda SSBs as aggressively. The Institute of Medicine (IOM) recommends that all SSBs be banned in schools [23,24], but this degree of restriction is rare. In 2010-11, 47% of high school students were in a district that banned soda in vending machines, but only 6% were in a district that banned all SSBs in vending machines [9]. In the same year, 87.8% of high school students nationwide reported having access to some type of SSB in school [15]. State laws that ban all SSBs in high schools are virtually nonexistent [25]. The Alliance School Beverage Guidelines, created from a partnership between the American Beverage Association, American Heart Association, and Clinton Foundation [26], permitted the sale of sports drinks and low-calorie soda (up to 10 calories per 8 ounces) in public and private high schools.

In the absence of more comprehensive policies, there is growing evidence of schools substituting soda with other SSBs. Studies in California [11] and Washington [27] reported that non-soda SSBs were widely available in schools even after sodas were removed. A U.S. national study also reported that student-reported access to SSBs did not decline if states only banned soda [12]. No study, to our knowledge, has examined whether similar substitution patterns have occurred in response to policies in other countries.

The notion of individuals substituting soda with other SSBs, in response to policy change, is frequently discussed but rarely studied. Other policy initiatives have been struck down on the rationale that people would simply substitute; when the New York City Board of Health attempted to limit SSB portion sizes, for example, state courts blocked the proposal in part because the limits would not apply to all SSBs in all locations [28]. Some experts have questioned the effectiveness of SSB taxes for similar reasons [29,30]. Simulation studies suggested that policies could reduce caloric intake even when substitution was taken into account [31-33], but these results were largely theoretical, not based on empirical data or randomized trials. Little research has directly examined whether policies that target soda are associated with higher consumption of non-soda SSBs.

This study was designed to analyze substitution patterns, particularly examining whether high school students consumed more sports drinks, energy drinks, and other SSBs if their state banned soda in school.

## Methods

This cross-sectional study linked student data from the National Youth Physical Activity and Nutrition Study (NYPANS), conducted by the Centers for Disease Control and Prevention (CDC) in spring 2010, with state laws regarding the sale of soda in school venues during the 2009-10 school year. The study was approved by the Institutional Review Boards of the University of Illinois at Chicago and the University of Texas Health Science Center at Houston.

## Student sample

NYPANS was designed to measure diet, physical activity, and sedentary behaviors, weight status, and environmental determinants of these measures in a nationally representative sample of 9^th^-12^th^ grade students [34]. Students were sampled using a 3-stage cluster sample design; the school response rate was 82%, the student response rate was 88%, and the overall response rate was 73%. In total, 10,887 public school students participated in NYPANS.

For the purpose of our study, students were excluded if they were missing data on self-reported vending machine access within school (n=691), SSB consumption (n=98), or other variables of interest (n=208), or if they were unsure if vending machines that sold SSBs were available in school (n=1,194). Students who were excluded did not differ from the study sample with respect to sex, weight status, vending machine access, or consumption of most sweetened and unsweetened beverages, but they were more likely to be racial/ethnic minorities (p<0.001), tended to be in lower grade levels (p<0.001), and tended to consume more diet soda, energy drinks, and coffee/tea than students in the study sample (p<0.001). The final study sample included 8,696 students in 27 states.

## Student-level measures

All student data that were utilized in this study were obtained using a written questionnaire completed in class. The NYPANS questionnaire was similar in format to the Youth Risk Behavior Survey that has been administered by the CDC in odd-numbered years since 1991 to study health risk behaviors in 9^th^-12^th^ grade students [35]. Our outcomes of interest were overall consumption of various SSBs other than regular soda, including diet soda, sports drinks, energy drinks, coffee/tea, and “other” SSBs (e.g., <100% fruit juice, flavored milk), each of which was measured using separate items. Students were asked to report the number of times they consumed each beverage in the previous 7 days; response options ranged from zero to “4 or more per day.” The questionnaire instructed students to include consumption from all locations. It should be pointed out that there was overlap between the questions for coffee/tea and “other SSBs,” as the latter included a list of examples that included “coffee drinks” and “sweetened tea.” It should also be pointed out that the question for coffee/tea included “any kind of tea,” not necessarily sweetened.

Students also reported whether the school had “a vending machine that students can use to purchase soda or pop, sports drinks, or fruit drinks that are not 100% juice, such as Coke, Gatorade, or Sunny Delight.” Hereafter, we use the term “vending machines” to refer specifically to this type of vending machine.

Other survey data that were utilized in this study were race/ethnicity, sex, grade, and home food access. Race and ethnicity were measured separately; for the purpose of this study, students were classified as non-Hispanic White, non-Hispanic Black, Hispanic, or non-Hispanic Other. Home food access was measured by asking students to report how often fruits and vegetables were available at home and how often chips/cookies/cakes were available at home. Both items included 5 responses ranging from “never” to “always.” For the purpose of this study, students were classified based on whether they usually or always had access to fruits and vegetables only, chips/cookies/cakes only, both, or neither.

## State data

Laws regarding the availability of soda in high school vending machines, school stores, and cafeterias (a la carte) in the 2009-2010 school year were obtained from the Westlaw and Lexis-Nexis legal research databases. Laws were double-coded by two trained coders and verified against secondary source state law data to ensure complete collection and coding interpretation. [36-38]. Laws for the three venues were analyzed separately and then combined to create one binary indicator of whether the state prohibited soda in all three venues. If a state banned soda only in specific venues (n=5), it was coded as a ‘0’ because this essentially meant that soda was not banned. These laws were compiled by the Bridging the Gap research program at the University of Illinois at Chicago.

To be clear, a school could adhere to state law by offering vending machines that sold SSBs other than soda. If students reported that vending machines were available, this was not an indication that schools were not adhering to state law, as schools could adhere by offering other SSBs. We explored studying whether laws were more effective if states banned SSBs other than soda, but no state in NYPANS had such restrictions on the high school level in 2009-10.

Analyses also utilized state data on Census region (Northeast, South, Midwest, or West) and median household income, which were obtained from the 2010 U.S. Census.

## Statistical analysis

All analyses used a zero-inflated negative binomial model due to the highly-skewed distributions of SSB consumption. For each SSB in this study, a large proportion of the sample consumed zero servings and a small proportion consumed a large quantity. For example, 42% of students consumed zero servings of sports drinks in the past 7 days, but 11% consumed 14 or more servings of sports drinks (mean=3.5, SD=6.0). The zero-inflated negative binomial model estimates both the log odds of students reporting zero servings and the log count of servings that they consumed [39].

In analyses of state soda laws, students were cross-classified based on whether their state banned the sale of soda in all school venues and whether their school had vending machines that sold other SSBs. This categorization scheme was chosen because soda bans that are similar, on paper, may have a different effect in practice if students still have access to other SSBs in vending machines. The reference group was students who had access to vending machines in school and resided in a state that allowed soda in school. Indicator variables were used to mark the other 3 groups: 1) students who had access to vending machines in a state that banned soda, 2) students with no access to vending machines in a state that allowed soda in school, and 3) students with no access to vending machines in a state that banned soda.

Each model controlled for race/ethnicity (reference group: non-Hispanic White), sex (reference group: boys), grade (continuous), home food access (reference group: access to fruits/vegetables only), Census region, and state median income (log-transformed) in the logistic portion of the model, and race/ethnicity, sex, grade, and home food access in the negative binomial portion of the model. Census region was modeled as a 3-category variable – South/Midwest (reference group), Northeast, West – because there were no Midwestern states in NYPANS that banned soda.

Analyses were conducted in the overall sample and then repeated by race/ethnicity and sex. This stratification was conducted because consumption of specific SSBs has been found to vary by race/ethnicity and sex [5,40]. All analyses utilized Stata, Version 13.

## Results

Table 1 displays descriptive statistics of the study sample, overall and by state/school measures of SSB access. Census region and racial/ethnic distributions differed considerably across categories of state laws. Specifically, no Midwestern states in the study banned soda. Due in part to the demographics of the Midwestern states, which tend to have a relatively large proportion of non-Hispanic White residents, students who resided in a state that did *not* ban soda were far more likely to be non-Hispanic White (64.4% vs. 44.9%) and less likely to be Hispanic (12.3% vs. 31.1%). Overall, students consumed an average of 5.4 servings of soda per week; among the SSBs that we analyzed in this study, ‘other SSBs’ such as <100% fruit juice were the most heavily consumed (4.1), followed by sports drinks (3.5). Students in states with no soda ban were more likely to report having access to SSB vending machines (84.0%), but such vending machines were still widely available in states with soda bans (68.9%).

**Table 1:** Summary statistics of study sample, National Youth Physical Activity and Nutrition Study (NYPANS), 2010 – overall and by state soda ban and school vending machine access

		Soda allowed in school		Soda banned in school	
Variable	Overall	Vending	No vending	Vending	No vending
*N*	8696	4452	969	2229	1046
*Gender (%)*					
Female	49.5	49.7	47.7	49.1	51.2
*Race/ethnicity (%)*					
Non-Hispanic White	58.1	64.8	62.6	47.2	39.6
Non-Hispanic Black	14.9	13.9	19.5	16.1	13.1
Hispanic	18.4	12.7	10.0	27.8	38.6
Non-Hispanic Other	8.6	8.6	8.0	8.9	8.7
*Grade (%)*					
9	26.6	26.8	28.0	26.1	24.9
10	25.4	25.9	27.4	27.4	21.3
11	24.6	24.0	26.1	26.1	26.6
12	23.4	23.4	20.4	20.4	27.2
*Census region (%)*					
South	37.7	34.5	30.1	47.0	42.8
Northeast	13.7	6.4	22.9	22.0	25.8
Midwest	26.5	41.1	30.0	0.0	0.0
West	22.1	18.0	17.0	31.0	31.4
*Weight status*					
Overweight (%)	18.1	17.4	19.6	18.2	19.7
Obese (%)	19.2	19.8	15.0	21.3	15.1
BMI percentile (mean)	67.8	67.5	66.1	69.9	66.3
*Beverage consumption – servings/week (mean, SD)*					
Soda	5.4 (7.2)	5.2 (6.7)	6.0 (7.7)	5.4 (7.9)	5.2 (7.9)
Diet soda	1.5 (4.2)	1.4 (3.9)	1.9 (5.1)	1.4 (4.3)	1.4 (4.5)
Sports drinks	3.5 (6.0)	3.2 (5.1)	3.7 (6.5)	4.1 (7.4)	3.8 (6.8)
Energy drink	1.1 (3.6)	1.0 (3.0)	1.5 (4.4)	1.3 (4.5)	1.2 (3.7)
Other SSBs (e.g., <100% fruit juice)	4.1 (6.0)	3.8 (5.5)	4.4 (6.9)	4.4 (6.4)	4.3 (6.3)
100% fruit juice	6.0 (7.4)	5.9 (6.8)	6.0 (7.6)	6.3 (8.5)	6.2 (7.8)
Coffee, coffee drinks, any type of tea	3.1 (5.4)	2.9 (4.9)	3.6 (6.5)	3.2 (5.8)	3.2 (5.6)
Water	15.4 (10.2)	15.3 (9.7)	15.2 (10.9)	16.0 (10.9)	15.0 (11.0)
*Other dietary behaviors (mean, SD)*					
Days of fast food/week	1.9 (1.7)	1.9 (1.6)	2.2 (1.9)	1.9 (1.8)	1.9 (1.9)
Cups of fruit/day	1.2 (1.1)	1.2 (1.0)	1.2 (1.1)	1.2 (1.2)	1.2 (1.2)
Cups of vegetables/day	0.9 (1.0)	0.9 (0.9)	1.0 (1.1)	1.0 (1.2)	0.9 (1.1)

Results from analyses of state soda laws and school vending machine access are presented in Table 2. Parameter estimates in Table 2 represent the difference in log odds of reporting zero servings (“logistic”) and difference in the log number of servings consumed in the past 7 days (“negative binomial”). Here, to make results more interpretable, we present the relative measures of consumption (e.g., RR=relative number of servings per week) that are calculated from the parameters in Table 2.

**Table 2:** Relative measures of students’ overall beverage consumption associated with state soda ban and school SSB vending machine access, based on zero-inflated negative binomial model

	Soda allowed in school						Soda banned in school					
	**Vending machine**			No vending machine			Vending machine			No vending machine		
Beverage	β	95% CI	p	β	95% CI	p	β	95% CI	p	β	95% CI	p
*Diet soda*												
Logistic	-	-	-	0.00	-0.22, 0.23	.97	0.15	-0.18, 0.48	.37	0.13	-0.19, 0.44	.43
Negative binomial	-	-	-	0.34	0.00, 0.68	.05	0.12	-0.08, 0.32	.24	0.10	-0.15, 0.36	.43
*Sports drinks*												
Logistic	-	-	-	0.21	-0.11, 0.52	.19	0.09	-0.29, 0.47	.63	-0.14	-0.52, 0.25	.48
Negative binomial	-	-	-	0.19	0.01, 0.37	.04	0.23	0.10, 0.35	.001	0.17	-0.02, 0.37	.08
*Energy drinks*												
Logistic	-	-	-	-0.07	-0.38, 0.23	.64	0.00	-0.21, 0.22	.98	-0.12	-0.67, 0.42	.65
Negative binomial	-	-	-	0.28	0.03, 0.53	.03	0.25	0.02, 0.47	.03	0.07	-0.27, 0.41	.70
*Coffee/tea*												
Logistic	-	-	-	0.06	-0.26, 0.39	.70	-0.03	-0.35, 0.28	.83	-0.29	-0.78, 0.20	.24
Negative binomial	-	-	-	0.24	0.02, 0.45	.03	0.16	0.01, 0.31	.03	0.14	-0.06, 0.33	.16
*Other SSB*												
Logistic	-	-	-	-0.11	-1.64, 1.42	.89	0.00	-0.97, 0.98	.99	-0.81	-2.51, 0.90	.35
Negative binomial	-	-	-	0.11	-0.07, 0.29	.24	0.16	0.00, 0.32	.05	0.14	0.01, 0.27	.03

State law bans the sale of soda in vending machines, school stores, and cafeterias (a la carte)

^†^ Vending machine that sells “soda or pop, sports drinks, or fruit drinks that are not 100% juice, such as Coke, Gatorade, or Sunny Delight”

^‡^ Adjusted for race/ethnicity, sex, grade, state median income, Census region, and home food access (negative binomial portion) and race/ethnicity, sex, grade, and home food access (logistic portion)

^δ^ Reference group

Consistently, state laws banning soda and vending machine access were not associated with the odds of reporting zero/no servings of SSBs. (As a reminder, the reference is students who had access to vending machines in a state that allowed soda.) Among SSB consumers, however, the quantity of consumption tended to be higher in 2 of the 3 comparison groups – 1) students with access to vending machines in a state that banned soda, and 2) students with no access to vending machines in a state that did not ban soda. In other words, SSB consumption tended to be higher if individual state or school restrictions on SSB access were in place, but usually not if both state and school restrictions were in place.

For example, students who had access to vending machines in a state that banned soda in schools (n=2229) consumed 1.25 times as many servings of sports drinks in the past 7 days (RR=1.25=exp(0.23), 95% CI: 1.11, 1.42). They also consumed more energy drinks (RR=1.29, 95% CI: 1.03, 1.62), coffee/tea (RR=1.18, 95% CI: 1.01, 1.36), and other SSBs (RR=1.16, 95% CI: 1.02, 1.32). Similarly, students who attended a school with no vending machines in a state that allowed soda (n=969) consumed more diet soda (RR=1.40, 95% CI: 1.00, 1.97), sports drinks (RR=1.22, 95% CI: 1.03, 1.45), energy drinks (RR=1.33, 95% CI: 1.03, 1.71), and coffee/tea (RR=1.27, 95% CI: 1.03, 1.56). In contrast, intake of SSBs was not elevated among students who attended a school with no vending machines in a state that banned soda (n=1046), with the exception of a slight positive association with the number of servings of ‘other SSBs’ such as <100% fruit juice.

Our stratified analyses suggested that state soda laws were associated with elevated intake of different SSBs in different sub-groups (Table 3). Only results from the negative binomial portion of the models are presented because, as in Table 2, there were no differences in the log odds of zero consumption. Notably, non-Hispanic Blacks consumed 1.70 times as many energy drinks (95% CI: 1.09, 2.65) and 1.49 times as many sports drinks (95% CI: 1.16, 1.92) if they resided in a state that banned soda in schools but had in-school access to vending machines. Boys in this category also consumed 1.41 times as many energy drinks (95% CI: 1.04, 1.92), whereas girls consumed 1.25 times as much coffee/tea (95% CI: 1.06, 1.47).

**Table 3:** Relative quantity of students’ overall beverage consumption associated with state soda ban and school SSB vending machine access, by race and gender

	**Soda allowed in school**						**Soda banned in school**					
	**Vending machine**			**No vending machine**			**Vending machine**			**No vending machine**		
	**RR**	**95% CI**	**p**	**RR**	**95% CI**	**p**	**RR**	**95% CI**	**p**	**RR**	**95% CI**	**p**
**Race/ethnicity**												
*Non-Hispanic Whites*												
Diet soda	-	-	-	1.26	0.78, 2.04	.34	0.95	0.74, 1.22	.69	0.79	0.57, 1.09	.15
Sports drinks	-	-	-	1.24	0.96, 1.60	.10	1.21	1.02, 1.44	.03	1.16	0.83, 1.60	.38
Energy drinks	-	-	-	1.10	0.83, 1.45	.51	1.09	0.81, 1.46	.57	0.86	0.61, 1.21	.37
Coffee/tea	-	-	-	1.22	0.92, 1.60	.17	1.25	1.01, 1.55	.04	1.19	0.94, 1.52	.15
Other SSBs	-	-	-	1.09	0.92, 1.29	.32	1.20	0.98, 1.47	.08	1.26	1.05, 1.51	.02
*Non-Hispanic Blacks*												
Diet soda	-	-	-	0.89	0.62, 1.27	.52	1.06	0.75, 1.50	.73	0.95	0.50, 1.82	.88
Sports drinks	-	-	-	0.97	0.78, 1.21	.81	1.49	1.16, 1.92	.003	1.31	0.82, 2.09	.25
Energy drinks	-	-	-	1.21	0.79, 1.85	.36	1.70	1.09, 2.65	.02	1.48	0.76, 2.89	.25
Coffee/tea	-	-	-	0.98	0.71, 1.36	.91	1.14	0.90, 1.46	.27	1.18	0.66, 2.13	.57
Other SSBs	-	-	-	1.02	0.81, 1.28	.85	1.28	1.09, 1.51	.004	1.25	0.92, 1.71	.15
*Hispanics*												
Diet soda	-	-	-	0.74	0.44, 1.26	.27	1.41	1.04, 1.92	.03	1.31	0.87, 1.98	.19
Sports drinks	-	-	-	1.88	0.80, 4.44	.15	1.20	0.88, 1.64	.25	1.26	0.99, 1.61	.06
Energy drinks	-	-	-	2.06	0.79, 5.40	.14	1.44	0.88, 2.36	.15	1.29	0.90, 1.85	.16
Coffee/tea	-	-	-	1.46	0.92, 2.31	.11	0.89	0.67, 1.16	.37	0.92	0.66, 1.30	.65
Other SSBs	-	-	-	0.98	0.66, 1.45	.92	1.03	0.79, 1.35	.81	1.06	0.87, 1.28	.58
**Gender**												
*Boys*												
Diet soda	-	-	-	1.47	0.90, 2.38	.12	1.39	1.11, 1.74	.005	1.15	0.77, 1.71	.48
Sports drinks	-	-	-	1.21	0.94, 1.56	.14	1.24	1.06, 1.44	.01	1.11	0.88, 1.40	.36
Energy drinks	-	-	-	1.51	1.08, 2.10	.02	1.41	1.04, 1.92	.03	0.94	0.67, 1.33	.73
Coffee/tea	-	-	-	1.46	1.14, 1.85	.003	1.08	0.86, 1.36	.51	0.93	0.73, 1.18	.52
Other SSBs	-	-	-	1.02	0.82, 1.28	.82	1.09	0.87, 1.36	.45	1.02	0.84, 1.23	.87
*Girls*												
Diet soda	-	-	-	1.19	0.83, 1.71	.35	0.95	0.72, 1.25	.71	1.11	0.83, 1.47	.48
Sports drinks	-	-	-	1.11	0.85, 1.44	.44	1.27	1.09, 1.47	.002	1.33	0.92, 1.94	.13
Energy drinks	-	-	-	1.10	0.72, 1.67	.66	1.11	0.73, 1.67	.62	1.31	0.71, 2.43	.39
Coffee/tea	-	-	-	1.06	0.79, 1.42	.68	1.25	1.06, 1.47	.01	1.33	0.99, 1.80	.06
Other SSBs	-	-	-	1.14	0.90, 1.45	.27	1.22	0.93, 1.59	.15	1.22	0.99, 1.50	.06

Estimated from zero-inflated negative binomial model; table only presents results from the negative binomial portion of the model. Adjusted for race/ethnicity (when stratifying by sex), sex (when stratifying by race/ethnicity), grade, state median income, Census region, and home food access (negative binomial portion), and race/ethnicity, sex, grade, and home food access (logistic portion)

^†^ State law bans the sale of soda in vending machines, school stores, and cafeterias (a la carte)

^‡^ Vending machine that sells “soda or pop, sports drinks, or fruit drinks that are not 100% juice, such as Coke, Gatorade, or Sunny Delight”

^δ^ Reference group

## Discussion

Understandably, sugar-sweetened soda has been the principal target of school nutrition policies in the U.S. in the past decade. Prior to the diffusion of school nutrition policies, sodas had been widely available in schools nationwide [15] and were consumed in large, unhealthy quantities by a large proportion of adolescents in the U.S. [2,4,5]. Policies have been very successful in removing soda from schools, as intended [11-14], but this study raises questions of how students may be compensating for such changes.

We found that students tended to consume more sports drinks, energy drinks, coffee/tea, and other SSBs if they resided in a state that only banned soda in schools. These trends were most apparent where the state banned soda at schools but students still had access to vending machines that sold sweetened beverages. The NYPANS measure of vending machines explicitly instructed students to include vending machines that sold any type of SSB, and nearly 70% of students in states with soda bans reported having access to such vending machines at school. Thus, it is plausible that schools in NYPANS adhered to laws by replacing soda with other SSBs, as other studies have reported [11,12,27]. We also found elevated SSB intake among students whose school did not have SSB vending machines but the state did not ban soda at school. The inverse association between vending machine access and SSB consumption may represent a different causal mechanism, such as students leaving campus if schools did not have vending machines and state laws were not actively requiring healthier beverages within school. We can only speculate about why these patterns were observed; additional research is needed to explore the mechanisms behind them.

Interestingly, SSB consumption was not elevated if both schools and states took action to reduce SSB access – i.e., states banned soda and schools did not offer vending machines. A recent study similarly reported that students without vending machines consumed more soda unless the state also banned soda in school [41]. Different factors may explain the null results that were observed when both schools and states restricted SSB access. One explanation is that the comprehensive effect on policy/environmental changes at the state and school level created healthier environments overall (e.g., access to healthier beverages). Previous research reported that the sale of unhealthy foods/beverages in elementary schools was often lowest when both state and local policies were enacted [13]. Low statistical power is another possible explanation for the null results, however, as the group with both state soda bans and no access to vending machines had a relatively small sample size.

Results also suggested that different sub-populations may have substituted soda with different SSBs. Non-Hispanic Blacks tended to consume more sports drinks and energy drinks, in particular, when the state banned soda; these results differ from racial/ethnic trends in SSB intake in the U.S. overall, as reported elsewhere [5]. The increase in energy drink consumption among Blacks is particularly concerning as health organizations continue to speak out against the harmful effects of energy drink consumption [19-21]. Energy drinks have no health benefits and include several additives that are understudied and unregulated [22]. They commonly contain 70-80 milligrams (mg) of caffeine per 8 ounces, more than twice the concentration in cola drinks; 20-ounce energy drink containers include up to 325 mg of caffeine, comparable to 3-4 cups of coffee [42].

Changes in industry marketing practices may have contributed to the results that we observed. In the U.S. in 2006, a coalition of food/beverage companies pledged to market “healthier dietary choices” to children <12 years old, but recent evidence suggests that companies’ marketing of unhealthy foods has shifted toward 12-17 year-olds [43]. Advertising spending on energy drinks alone increased from $120 million to $164 million from 2008 to 2010 [18]. Research has also shown that beverage companies’ marketing is often targeted at Black and Hispanic youth [18]. More research is needed to determine if changes in marketing practices have contributed to high consumption of SSBs that were observed in this study and in the general population [5].

These results underscore the limited scope of existing school beverage guidelines at the high school level. In the 2009-2010 school year, when NYPANS was conducted, 14 of 50 states in the U.S. met IOM recommendations to ban all SSBs at the elementary school level, but Connecticut was the only state that met IOM recommendations for high schools [25]. High school students are a population whose SSB preference has shifted toward non-soda SSBs in recent years [5]. This shift may be a direct consequence of policies that targeted soda, of increased marking of other SSBs, or it may be a coincidence. Regardless, policies need to shift accordingly to improve the overall quality of adolescents’ beverage intake and thereby reduce the health risks that are associated with overall sweetened beverage consumption. Policies that restrict SSB availability should also be accompanied by initiatives that promote low-cost alternatives such as filtered water while recognizing that beverage choices are commonly based on taste and price, not health [44].

As with any observational study, we cannot conclude that laws or vending machine access were the causes of elevated SSB intake. States may have banned soda, in part, because of students’ elevated SSB intake, which would partially explain the associations that we observed. We could not control for unmeasured student-level variables such as socioeconomic status (SES), either. Our analyses of vending machines were further limited because students were not asked which specific SSBs were available in vending machines, nor were students asked where they were consuming these beverages or whether SSBs were available in other school venues. Therefore, we cannot ascertain if schools were substituting soda with other SSBs or students were simply obtaining SSBs elsewhere. We should also point out that, among the three comparison groups in our analysis, the sample size was largest for the category of students who had access to vending machines in a state that banned soda; statistical power was more limited in the other two groups. NYPANS was also designed to be representative at the national, not state, level.

Finally, we did not analyze specific measures of nutrient intake, a topic that should be explored further in future research. The overall impact of SSB substitution on students’ nutrient intake, weight status, and cardio-metabolic health has not been studied extensively and is difficult to project due to variance in serving sizes and compositional differences between beverages and brands. The energy and sugar content of energy drinks and fruit drinks can vary enormously by brand and variety [45]. Sports drinks contain 50-90% as much energy per ounce as soda [46], but are commonly sold in 32-ounce containers (in contrast to the typical 12-ounce soda can or 20-ounce soda bottle.) Furthermore, traditional SSB measures do not fully capture beverages that are calorically sweetened after purchase, but recent methods have been designed to account for this [47]. Careful research is needed to determine how SSB substitution behaviors translate to health outcomes when these variables are taken into account.

## Conclusions

Our study raises concerns about whether policymakers’ focus on soda may have unintended negative consequences at the high school level, as laws that ban soda were associated with higher intake of other SSBs when vending machines were still available. We cannot prove causality, but the elevated SSB intake suggests that state soda bans were, at best, not addressing the beverage consumption patterns of the adolescent populations in these states. Adolescents’ SSB preferences have shifted in the U.S. overall, but high school SSB policies have not followed suit even though high school is a period when children tend to consume the most SSBs. Existing policies may need to be expanded to restrict the sale of all SSBs, in accordance with IOM recommendations [23,24], and ensure that students are able to access low-cost healthier alternatives in school. Additional longitudinal research is needed to determine whether more comprehensive SSB restrictions can reduce consumption of all SSBs.

## Competing interests

The authors declare that they have no competing interests.

## Author contributions

DT conceived and designed the study, oversaw the statistical analyses, and led the writing of the manuscript. JC and FC led the policy data collection and contributed to the study conceptualization. RV conducted the statistical analyses. JC, RV, SK, and FC contributed to interpretation of results and drafting of the manuscript. All authors read and approved the final document.

## Declarations

This article has been published as part of *International Journal of Behavioral Nutrition and Physical Activity* Volume 12 Supplement 1, 2015: The science of childhood obesity: an individual to societal framework. The full contents of the supplement are available online at http://www.ijbnpa.org/supplements/12/S1. Publication costs for this supplement were funded by the Michael & Susan Dell Foundation.
